# The Psychometric Properties of the Resilience Scale (RS-14) in Lithuanian Adolescents

**DOI:** 10.3389/fpsyg.2021.667285

**Published:** 2021-05-21

**Authors:** Paulina Zelviene, Lina Jovarauskaite, Inga Truskauskaite-Kuneviciene

**Affiliations:** Center for Psychotraumatology, Institute of Psychology, Vilnius University, Vilnius, Lithuania

**Keywords:** resilience, adolescence, socio-emotional problems, latent class analysis, confirmatory factor analysis, measurement invariance, validity

## Abstract

In the current study, we provided the evidence of satisfactory validity of the RS-14 scale in the Lithuanian adolescents’ sample (*N* = 1299; *M*_age_ = 14.24; *SD*_age_ = 1.26), based on its internal structure, and relations to other variables. The results of the study indicated an acceptable model fit for a single-factor structure of the scale with a high internal consistency (McDonald’s omega = 0.89). We also confirmed the scalar measurement invariance across groups of adolescents in terms of their age (i.e., early and middle adolescence) and mental health profile as well as partial scalar gender invariance. Adolescents characterized by high levels of socio-emotional problems reported lower levels of resilience, in comparison to adolescents that reported low levels of socio-emotional problems. However, the data indicated that adolescents from emotional problems and behavioral problems groups cannot be differentiated with the RS-14 scale.

## Introduction

The growing interest in resilience in psychological research is closely related to a high demand for its measurement tools. The nature of resilience is complex because it involves interaction among biological, psychological, social, and cultural factors which determine the initial response to stress (Masten et al., 2021). Furthermore, resilience could be defined as a stable personality characteristic, a dynamic process, or an outcome (Southwick et al., 2014). Hence the measurement of resilience requires theory-driven, age- and culture-specific tools with the relevant psychometric characteristics. The Resilience Scale (RS-14) is a brief theory-based measure that covers the key elements of resilience. The theoretical background of this scale is rooted in the assumption that the core of resilience can be characterized through purpose, perseverance, equanimity, self-reliance, and existential aloneness (Wagnild, 2009). The RS-14 is measuring trait resilience which was found to be associated with mental health across multiple studies (Hu et al., 2015). If the core of resilience is strong, the possibility to function better increases along with learning from the experience or even personal growth. On the contrary, the weaker core of resilience increases the possibility of giving up or feeling desperation.

The validity and reliability of the RS-14 scale were addressed in the number of cross-cultural studies in which Italian (Callegari et al., 2016), Finnish (Losoi et al., 2013), Greek (Ntountoulaki et al., 2017), Japanese (Nishi et al., 2010) versions of the scale were investigated in adult populations. The results of the studies indicated a good internal consistency of the RS-14, the value of Cronbach alpha ranging from 0.76 to 0.96 (Miroševič et al., 2019). However, the results regarding the internal structure of the scale were mixed. Most of the studies confirmed a single-factor structure of the RS-14 (e.g., Nishi et al., 2010; Ntountoulaki et al., 2017), whereas other studies indicated a poor model fit for a single-factor structure (Losoi et al., 2013) or found multidimensional structure, i.e., three-factor solution (Callegari et al., 2016) of this scale.

The RS-14 scale was also applied to evaluate adolescents’ resilience in several studies. Pritzker and Minter (2014) indicated a good internal consistency (Cronbach alpha were 0.91 and 0.96 and) and a one-factor solution of RS-14. More recently, Surzykiewicz et al. (2019) investigated the validity and reliability of RS-14 in problematic, non-problematic samples of adolescents as well as young adults. The results of the study indicated a single-factor structure of the scale with a good internal consistency (Cronbach α = 0.85), and test-retest reliability (*r* = 0.88). However, the study conducted by Chung et al. (2020) confirmed a good internal consistency (Cronbach α = 0.86) and reliability (*r* = 0.84) of RS-14 in Chinese adolescents, but the scale contained two dimensions, namely, personal competence, and acceptance of self and life.

The evidence of the validity of RS-14 based on relations to other variables was also addressed across the studies as a part of psychometric characteristics aimed to predict psychological difficulties. In general, as the meta-analyses or systematic reviews show, resilience is related to better mental health and functioning (van der Meulen et al., 2020), the quality of life, lower rates of anxiety, and depression (Miroševič et al., 2019). The results of the studies also indicated that emotional, and behavioral problems were associated with lower resilience rates (Huang et al., 2019). Furthermore, in the sample of adolescents with chronic illness higher rates of resilience were associated with the lower scores of pain, physical disability, symptoms severity, and higher rates of energy, quality of life, related to health (Gmuca et al., 2019). The study conducted by Hildebrand et al. (2019) showed significant links between social support and resilience in children and adolescents.

The RS-14 scale is a widely used measure to assess the ability to overcome adversity in adolescence, however, the internal structure of the scale seems to vary across different contexts. Therefore, the current study aimed to investigate the psychometric properties of the RS-14 scale in a large sample of Lithuanian adolescents. First, following the theoretical conceptualization of the unidimensional nature of resilience reflected in the RS-14 measure (Wagnild, 2009), we sought to collect the validity evidence based on a single-factor internal structure of the scale as well as its reliability. Second, to collect the evidence of validity based on relations to other variables (American Educational Research Association (AERA) et al., 2014) we applied both variable-oriented and a person-oriented approach and investigated the links between resilience and indicators of socio-emotional functioning as well as the role of resilience in differentiating distinct groups of adolescents with different profiles of socio-emotional functioning. Based on previous studies (e.g., Huang et al., 2019), we expected that the correlations between resilience and the indicators of socio-emotional functioning would be low-to-moderate. Also, as it is theoretically conceptualized that resilience plays an important role as a protective factor in the development of mental health problems in adolescents (e.g., Masten, 2007), we hypothesized that adolescents with low levels of socio-emotional problems would have higher resilience rates.

## Method

### Participants and Procedures

The data from the first wave of the currently ongoing longitudinal study Stress and Resilience in Adolescence (STAR-A) was used in the current study. The study is led by the Center for Psychotraumatology of Vilnius University in Lithuania. The study design was developed in cooperation with the Norwegian Center for Violence and Traumatic Stress Studies (NKVTS). The STAR-A study was approved by the Ethics Committee for Psychological Research at Vilnius University.

The data were collected in public schools from four different regions in Lithuania in 2019 using self-report measures. Written informed consent from at least one parent was obtained prior to data collection. Data were collected in person by a trained research team. Before starting the data collection, adolescents were informed about the aims and procedures of the study as well as the possibility to decline participation in the study. No incentives were offered for participation to either parents or adolescents.

In total, 1299 adolescents (56.6% girls, *M*_age_ = 14.24; *SD*_age_ = 1.26) participated in the current study. The nationality in the sample was predominantly Lithuanian (92.7%). Most of the adolescents (72.0%) were from two-parent families; 69.3% of adolescents reported that either one or both parents have a university degree; 40.0% of adolescents were from families, experiencing financial difficulties. A detailed description of the sample and study procedures were published previously (Kazlauskas et al., 2020; Zelviene et al., 2020).

### Measures

**The Resilience Scale (RS-14).** The RS-14 scale comprises 14 items measuring a single construct of psychological resilience (Wagnild, 2009). Each item is rated on a 7-point Likert scale. Previous research has shown good reliability of the scale both in adult (Ahern et al., 2006) and adolescent (e.g., Pritzker and Minter, 2014) samples. The Lithuanian version of the scale has been validated in the adult sample (Mažulytė et al., 2014).

**The Strengths and Difficulties Questionnaire (SDQ)**. The SDQ was used to measure emotional and behavioral problems in adolescence (Goodman, 2001). The SDQ comprises 25 items, divided into five scales, representing five psychosocial functioning dimensions, five items in each. Problems are reflected by the scales of *hyperactivity/inattention, emotional symptoms*, *conduct problems*, and *peer relationship problems*; positive psychosocial functioning is reflected in the scale of *prosocial behavior*. The SDQ is widely used globally and has shown acceptable reliability and validity across many cultures (Goodman, 2001). The SDQ has been validated in a representative sample of Lithuanian children and adolescents with Cronbach’s alpha of the self-report version of the full scale equal to0.72 and acceptable model fit of five-factor confirmatory factor analysis (Gintiliene et al., 2004; Lesinskiene et al., 2018).

### Data Analysis

To collect the evidence of validity based on the internal structure of the Resilience Scale (RS-14), the single-factor Confirmatory Factor analysis (CFA) was conducted in a full study sample. To test whether the scale is suitable for use among both genders and different age groups, we conducted the measurement invariance test by gender (female vs. male) and age (early (12–14 years old) adolescents vs. middle (15–16 years old) adolescents). First, we compared the configural CFA models, testing the basic model structure without any between-group invariance constraints on estimated parameters, with the metric model, in which the factor loadings were constrained to be equal across groups, and the scalar model, in which item intercepts were also constrained to be equal across groups. Model comparisons were conducted by examining the changes in fit indices, where ΔCFI ≥ 0.010 supplemented by ΔRMSEA ≥ 0.015 were indicative of the significant difference between models (e.g., Chen, 2007). To test the reliability of the scale, we computed McDonald’s omega reliability coefficients (McDonald, 1978). The McDonald’s omega is interpreted the same as Cronbach’s alpha (Geldhof et al., 2014).

To investigate the evidence of the validity of the RS-14 scale based on relations to other variables, we first applied the variable-oriented approach and correlated the sum scores of the full scale with the sum scores of subscales of the Strengths and Difficulties Questionnaire (SDQ), in particular, emotional symptoms, conduct problems, hyperactivity/inattention, peer relationship problems, and prosocial behavior. Further, we applied the person-oriented approach and compared the levels of resilience among adolescents with different profiles of socio-emotional problems. To do that, first, we used the data-driven approach to identify the subgroups of the participants in terms of socio-emotional problems and conducted the Latent Class Analysis (LCA; Muthén and Muthén, 2000). For LCA, we used the sum scores of prosocial behavior, hyperactivity/inattention, emotional symptoms, conduct problems, and peer relationship problems. We used several criteria to decide on a final number of latent classes (Muthén and Muthén, 2000). In particular, Akaike Information Criterion (AIC) and Bayesian Information Criterion (BIC) statistic for a solution with k classes should be lower than for a solution with k - 1 class; a statistically significant *p*-value of the adjusted Lo-, Mandel-, and Rubin test, which compares improvement in fit between neighboring class solutions, determining improvement in fit through the inclusion of an additional class; Entropy score, with the values equal or above0.70 indicative of accurate classification with higher values representing a better fit; the number of participants in the smallest class which should be not lower than 5% of the study sample. Then, to compare the distinguished groups in terms of the resilience levels, we first established the measurement invariance across groups and then compared the latent means of the resilience among the groups by constraining the mean in the reference group to 0 and freeing the mean in the comparison groups. When comparing the latent means, a significant mean in a comparison group indicates a significant difference among groups.

The model fits in CFA analyses were evaluated by using the Comparative Fit Index (CFI), the Tucker–Lewis Index (TLI), and the Root Mean Square Error of Approximation (RMSEA), following the goodness of fit recommendation provided by Kline (2011). Namely, CFI/TLI values higher than 0.90 indicated an acceptable fit, and values higher than 0.95 represented a good fit; RMSEA values below 0.08 indicated an acceptable fit, and values less than 0.05 suggested a good fit. The descriptive and correlational analyses were conducted using SPSS 24, for all other analyses the Mplus 8.2 version (Muthén and Muthén, 1998-2017) was used.

## Results

### The Evidence of Validity Based on Internal Structure

The results of initial confirmatory factor analysis (CFA) yielded rather unacceptable model fit [χ^2^(77) = 693.21, *p* < 0.001, CFI/TLI = 0.870/0.846, RMSEA [90% CI] = 0.079 [0.073;0.084], SRMR = 0.052]. Therefore, based on modification indices, ten correlations of error pairs were added one by one. The final CFA confirmed the one-factor structure of the Resilience scale in the adolescents’ sample with the acceptable model fit [χ^2^(67) = 303.72, *p* < 0.001, CFI/TLI = 0.950/0.932, RMSEA [90% CI] = 0.052 [0.046;0.058], SRMR = 0.035]. The standardized factor loadings of the items ranged from 0.43 to 0.76 (see Supplementary Table 1). The results of the measurement invariance among gender and age groups are presented in Table 1. The scalar measurement invariance by age and the partial scalar measurement invariance by gender were established by allowing for the intercepts of three items of the RS-14 scale (i.e., item nr. 3, 4, and 12) to vary across gender groups. Additionally, the reliability of the scale in a full study sample was found to be high (McDonald’s omega = 0.89). The high consistency was confirmed among girls (McDonald’s omega = 0.90) and boys (McDonald’s omega = 0.88) as well as early adolescents (McDonald’s omega = 0.89) and middle adolescents (McDonald’s omega = 0.89).

**TABLE 1:** Results of measurement invariance tests by gender, age, and socio-emotional functioning froups.

	Model fit indices			Model comparisons	
	χ^2^(df)	CFI	RMSEA [90% CI]	ΔCFI	ΔRMSEA
**Gender**					
Configural	381.539 (134)	0.948	0.053 [0.047;0.060]		
Metric	402.607 (147)	0.946	0.052 [0.046;0.058]	0.002	0.001
Scalar	583.988 (160)	0.911	0.064 [0.058;0.070]	0.035	0.012
Partial scalar	430.754 (154)	0.942	0.053 [0.047;0.059]	0.004	0.001
**Age**					
Configural	383.935 (134)	0.948	0.054 [0.047;0.060]		
Metric	400.320 (147)	0.947	0.052 [0.045;0.058]	0.001	0.002
Scalar	431.969 (160)	0.944	0.051 [0.045;0.057]	0.003	0.001
**SDQ classes**					
Configural	543.307 (268)	0.942	0.056 [0.049;0.063]		
Metric	599.421 (307)	0.939	0.054 [0.048;0.061]	0.003	0.002
Scalar	681.277 (346)	0.930	0.055 [0.049;0.061]	0.009	0.001

*χ^2^, chi-square; df, degrees of freedom; CFI, comparative fit index; RMSEA, root mean square error of approximation; CI, confidence interval; Δ, change in the parameter.*

### Evidence of Validity Based on Relations to Other Variables

The results of the correlation analyses are presented in Table 2. The results indicated low-to-moderate links between resilience and the indicators of socio-emotional functioning in adolescence in the full sample as well as for both genders and among different age groups. Additionally, based on latent man analysis, we found no differences between the two genders in terms of resilience levels (Latent mean = 0.08, *p* = 0.79), when early adolescents reported lower levels of resilience, compared to middle adolescents (Latent mean = 0.09, *p* = 0.049).

**TABLE 2:** Descriptive statistics and correlation coefficients with resilience.

	Female (*n* = 735)		Male (*n* = 564)		Early adolescents (*n* = 624)		Middle adolescents (*n* = 675)		Total sample (*N* = 1299)	
	
	*M* (*SD*)	*r*	*M* (*SD*)	*r*	*M* (*SD*)	*r*	*M* (*SD*)	*r*	*M* (*SD*)	*r*
Resilience	71.69 (13.93)	–	72.98 (13.09)	–	71.3 (13.77)	–	73.11 (13.4)	–	72.24 (13.6)	–
Prosocial behaviors	7.74 (1.84)	0.39	6.53 (2.10)	0.33	7.11 (2.05)	0.40	7.31 (2.05)	0.27	7.22 (2.05)	0.33
Hyperactivity/inattention	3.62 (2.13)	−0.30	3.75 (2.04)	−0.38	3.73 (2.12)	−0.37	3.64 (2.08)	−0.29	3.69 (2.1)	−0.33
Emotional symptoms	3.64 (2.49)	−0.45	2.06 (1.98)	−0.41	2.81 (2.37)	−0.41	3.1 (2.45)	−0.44	2.96 (2.42)	−0.42
Behavioral problems	2.46 (1.46)	−0.22	2.65 (1.48)	−0.23	2.72 (1.56)	−0.29	2.39 (1.39)	−0.14	2.55 (1.48)	−0.22
Peer relationship problems	2.19 (1.75)	−0.26	2.24 (1.73)	−0.38	2.31 (1.78)	−0.32	2.14 (1.71)	−0.29	2.22 (1.74)	−0.31
Total SDQ	19.65 (5.15)	−0.36	17.23 (5.19)	−0.36	18.67 (5.24)	−0.38	18.56 (5.36)	−0.34	18.61 (5.3)	−0.36

*M, mean; SD, standard deviation; r, Pearson correlation coefficient. All correlation coefficients are significant at p < 0.001.*

**FIGURE 1:**
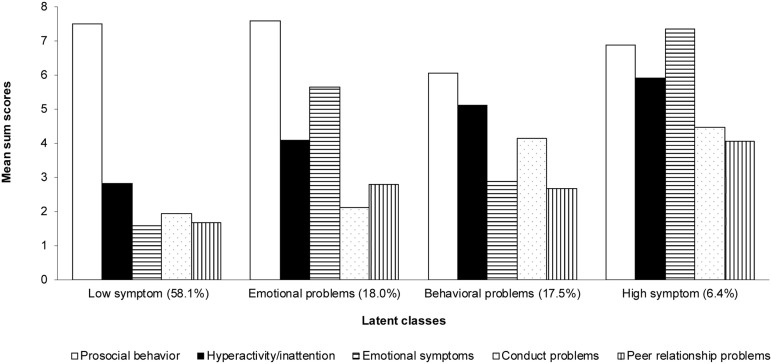
Latent classes; based on socio-emotional functioning mean sum scores (*N* = 1299).

The results of LCA analyses indicated that the four-classes solution fitted the data best (see Supplementary Table 2). The classes, based on the levels of prosocial behavior, hyperactivity/inattention, emotional symptoms, conduct problems, and peer relationship problems are presented in Figure 1. We found that, in our sample, over a half of adolescents (58.1%) were represented in a group with relatively low levels of all socio-emotional problems and we labeled this class as *low-symptom* (McDonald’s omega = 0.88). The second most prevalent group (18.0%) was characterized by relatively higher levels of emotional symptoms and hyperactivity/inattention with relatively lower levels of conduct problems and peer relationship problems; we labeled this class as *emotional-problems* (McDonald’s omega = 0.89). The third class (17.5%) was characterized by relatively higher levels of conduct problems and hyperactivity/inattention with relatively lower levels of emotional symptoms and peer relationship problems; we labeled this class as *behavioral-problems* (McDonald’s omega = 0.88). The last class, representing the lowest proportion of the adolescents in the sample (6.4%) was characterized by relatively high levels of all socio-emotional problems and we labeled this class as *high-symptom* (McDonald’s omega = 0.88).

The scalar measurement invariance was established among the classes (see Table 1). The subsequent comparison of latent means among the classes indicated that the adolescents with different profiles of socio-emotional problems were found to be characterized by different levels of resilience. In particular, we found that *low-symptom* group (*M* = 75.16, *SD* = 12.48) reported higher levels of resilience, compared to *emotional-problems* group (*M* = 69.47, *SD* = 13.81; latent mean = −0.37, *p* < 0.001), *behavioral-problems* group (*M* = 68.44, *SD* = 13.11; latent mean = −0.39, *p* < 0.001), and *high-symptom* group (*M* = 63.67, *SD* = 14.87; β = −0.71, *p* < 0.001). In contrast, the *high-symptom* group reported lower levels of resilience, compared to *emotional-problems* group (latent mean = 0.35, *p* = 0.003) and *behavioral-problems* group (latent mean = 0.32, *p* = 0.006). No differences were found between the *emotional-problems* and the *behavioral-problems* groups (latent mean = −0.03, *p* = 0.718).

## Discussion

The current study adds to the growing body of literature confirming the RS-14 scale (Wagnild, 2009) to be an acceptably valid tool for assessing psychological resilience in adolescence (Pritzker and Minter, 2014; Miroševič et al., 2019). Overall, we found that fourteen items comprising the RS-14 scale make up the single construct of resilience, as it was also reported in previous adolescent studies (e.g., Pritzker and Minter, 2014; Surzykiewicz et al., 2019). Our study indicated high reliability of the RS-14 scale (McDonald’s omega = 0.89) and it replicates the results of the past international studies in which the RS-14 scale was translated into other languages (e.g., Callegari et al., 2016). Thus, the current study contributes to the theoretical conceptualization of resilience by providing evidence for a univariate trait resilience construct (Hu et al., 2015).

Only a small number of previous studies addressed the measurement invariance of the RS-14 scale. In our study configural, metric, and scalar measurement invariance testing indicated that the RS-14 measure can be used for assessing resilience among early and middle adolescent age groups as well as among adolescents with different levels of socio-emotional problems. However, the gender invariance test indicated slightly problematic RS-14 scale use among two genders. Also, we found high reliability of the RS-14 scale both in non-risk and mental health risk group samples, confirming that the scale to be suitable for use in clinical adolescent samples (Surzykiewicz et al., 2019).

Finally, we revealed that the RS-14 scale is suitable for differentiating adolescents with different mental health profiles, as the level of resilience differed among socio-emotional problems groups. In particular, higher resilience was found to be characteristic among adolescents with low levels of socio-emotional problems in comparison to other mental health risk groups, including emotional, behavioral, and high levels of socio-emotional problems groups. In general, the findings of the current study are in line with previous research, indicating that resilience predicts different levels of mental health (Miroševič et al., 2019; van der Meulen et al., 2020). More particularly, resilience was confirmed to be negatively linked to socio-emotional problems when measured with the same scales (e.g., Huang et al., 2019). The results of the current study showed that the RS-14 scale could be used for differentiating adolescents that are most at risk in terms of mental health problems, reporting the combination of internalizing and externalizing symptoms, in comparison to those who reported high levels of emotional or behavioral problems. However, based on our findings, the RS-14 scale is not particularly useful in differentiating adolescents with different profiles of mental health problems, as the levels of resilience did not differ among adolescents with predominantly emotional or behavioral problems.

Although the current study was conducted in a relatively large sample of both early and middle adolescents, the present findings should be seen in the light of both strengths and limitations. First, the study was conducted in a non-representative sample of Lithuanian adolescents. Second, the study was cross-sectional, therefore the test-retest reliability could not have been established. Third, although we used both variable-oriented and person-oriented approaches, the evidence of validity based on relations to other variables should be further explored by using the longitudinal study design, as it would allow evaluating the links between resilience and adolescent mental health over time. Moreover, future longitudinal studies would benefit from addressing the questions, whether the RS-14 scale may capture changes in resilience over time. Finally, it should be noted that we managed to establish a single factor structure of the RS-14 scale only by adding correlations between the error pairs of the items. Also, some factor loadings were rather low. This could indicate that not all items may work as well as expected and, presumably, the shorter version of the scale could be developed by removing items with lower factor loadings. Despite these limitations, the findings of the current study confirm that the RS-14 scale is a psychometrically acceptable brief measure and could be used for assessing the adolescents’ adaptation to adverse life experiences in research, educational, and clinical settings in Lithuania.

## Data Availability Statement

The raw data supporting the conclusions of this article will be made available by the authors, without undue reservation.

## Ethics Statement

The studies involving human participants were reviewed and approved by Vilnius University Psychological Research Ethics Committee. Written informed consent to participate in this study was provided by the participants’ legal guardian/next of kin.

## Author Contributions

PZ: research planning, data collection, comments and improvements of the first draft. LJ: research planning and writing up the first draft. IT-K: research planning, data analytic plan, data analysis, and writing up the first draft. All authors contributed to the article and approved the submitted version.

## Conflict of Interest

The authors declare that the research was conducted in the absence of any commercial or financial relationships that could be construed as a potential conflict of interest.
