# Supplementary material for: The Psychometric Properties of the Resilience Scale (RS-14) in Lithuanian Adolescents
Source: Front Psychol. 2021 May 21;12:667285. doi: 10.3389/fpsyg.2021.667285 (PMC8175371; doi:10.3389/fpsyg.2021.667285)
Supplement: Supplementary file 1 [file Table_1.DOCX]

**Table S1**

*Standardized factor loadings of the RS-14 items*

| Resilience (RS-14) | *FL* | *SE* | *p* |
| --- | --- | --- | --- |
| Item1 | .634 | .025 | <.001 |
| Item2 | .638 | .021 | <.001 |
| Item3 | .531 | .024 | <.001 |
| Item4 | .702 | .019 | <.001 |
| Item5 | .641 | .021 | <.001 |
| Item6 | .655 | .023 | <.001 |
| Item7 | .496 | .027 | <.001 |
| Item8 | .481 | .027 | <.001 |
| Item9 | .510 | .027 | <.001 |
| Item10 | .425 | .030 | <.001 |
| Item11 | .701 | .018 | <.001 |
| Item12 | .519 | .026 | <.001 |
| Item13 | .702 | .019 | <.001 |
| Item14 | .764 | .015 | <.001 |

*Note.* FL = standardized factor loading, SE = standard error.

**Table S2**

*Model Fit Indices of Latent Class Analyses*

| Solution | Loglikelihood | AIC | BIC | Entropy | LMR-A  *p*-value | Smallest class count (%) |
| --- | --- | --- | --- | --- | --- | --- |
| 1 class | -13446.86 | 26913.72 | 26965.40 | - | - | - |
| 2 classes | -13175.87 | 26383.74 | 26466.42 | .674 | < .001 | 28.37% |
| 3 classes | -13093.04 | 26230.07 | 26343.76 | .740 | .013 | 18.82% |
| **4 classes** | **-13031.82** | **26119.65** | **26264.34** | **.744** | **.033** | **6.85%** |
| 5 classes | -12972.22 | 26012.44 | 26188.15 | .751 | .087 | 7.09% |

*Note*. AIC = Akaike Information Criterion; BIC = Bayesian Information Criterion; LMR-A = Lo-Mendell-Rubin Adjusted Likelihood Ratio Test (LMR-A). Best fitting solution is in bold.
